# Advances in biomaterials for oral-maxillofacial bone regeneration: spotlight on periodontal and alveolar bone strategies

**DOI:** 10.1093/rb/rbae078

**Published:** 2024-07-04

**Authors:** Nayun Li, Jinyu Wang, Guangxia Feng, Yuqing Liu, Yunsong Shi, Yifan Wang, Lili Chen

**Affiliations:** Department of Stomatology, Union Hospital, Tongji Medical College, Huazhong University of Science and Technology, Wuhan 430022, China; School of Stomatology, Tongji Medical College, Huazhong University of Science and Technology, Wuhan 430030, China; Hubei Province Key Laboratory of Oral and Maxillofacial Development and Regeneration, Union Hospital,Tongji Medical College, Huazhong University of Science and Technology, Wuhan 430022, China; Hubei Engineering Research Center for Oral and Maxillofacial Medical Devices and Equipment, Union Hospital, Tongji Medical College, Huazhong University of Science and Technology, Wuhan 430022, China; School of Stomatology, Tongji Medical College, Huazhong University of Science and Technology, Wuhan 430030, China; Hubei Province Key Laboratory of Oral and Maxillofacial Development and Regeneration, Union Hospital,Tongji Medical College, Huazhong University of Science and Technology, Wuhan 430022, China; Hubei Engineering Research Center for Oral and Maxillofacial Medical Devices and Equipment, Union Hospital, Tongji Medical College, Huazhong University of Science and Technology, Wuhan 430022, China; Department of Stomatology, Union Hospital, Tongji Medical College, Huazhong University of Science and Technology, Wuhan 430022, China; School of Stomatology, Tongji Medical College, Huazhong University of Science and Technology, Wuhan 430030, China; Hubei Province Key Laboratory of Oral and Maxillofacial Development and Regeneration, Union Hospital,Tongji Medical College, Huazhong University of Science and Technology, Wuhan 430022, China; Hubei Engineering Research Center for Oral and Maxillofacial Medical Devices and Equipment, Union Hospital, Tongji Medical College, Huazhong University of Science and Technology, Wuhan 430022, China; Department of Stomatology, Union Hospital, Tongji Medical College, Huazhong University of Science and Technology, Wuhan 430022, China; School of Stomatology, Tongji Medical College, Huazhong University of Science and Technology, Wuhan 430030, China; Hubei Province Key Laboratory of Oral and Maxillofacial Development and Regeneration, Union Hospital,Tongji Medical College, Huazhong University of Science and Technology, Wuhan 430022, China; Hubei Engineering Research Center for Oral and Maxillofacial Medical Devices and Equipment, Union Hospital, Tongji Medical College, Huazhong University of Science and Technology, Wuhan 430022, China; School of Stomatology, Tongji Medical College, Huazhong University of Science and Technology, Wuhan 430030, China; Hubei Province Key Laboratory of Oral and Maxillofacial Development and Regeneration, Union Hospital,Tongji Medical College, Huazhong University of Science and Technology, Wuhan 430022, China; Hubei Engineering Research Center for Oral and Maxillofacial Medical Devices and Equipment, Union Hospital, Tongji Medical College, Huazhong University of Science and Technology, Wuhan 430022, China; School of Stomatology, Tongji Medical College, Huazhong University of Science and Technology, Wuhan 430030, China; Hubei Province Key Laboratory of Oral and Maxillofacial Development and Regeneration, Union Hospital,Tongji Medical College, Huazhong University of Science and Technology, Wuhan 430022, China; Hubei Engineering Research Center for Oral and Maxillofacial Medical Devices and Equipment, Union Hospital, Tongji Medical College, Huazhong University of Science and Technology, Wuhan 430022, China; Department of Stomatology, Union Hospital, Tongji Medical College, Huazhong University of Science and Technology, Wuhan 430022, China; School of Stomatology, Tongji Medical College, Huazhong University of Science and Technology, Wuhan 430030, China; Hubei Province Key Laboratory of Oral and Maxillofacial Development and Regeneration, Union Hospital,Tongji Medical College, Huazhong University of Science and Technology, Wuhan 430022, China; Hubei Engineering Research Center for Oral and Maxillofacial Medical Devices and Equipment, Union Hospital, Tongji Medical College, Huazhong University of Science and Technology, Wuhan 430022, China

**Keywords:** oral-maxillofacial bone, biomedical materials, dental materials, bone repair, periodontal regeneration

## Abstract

The intricate nature of oral-maxillofacial structure and function, coupled with the dynamic oral bacterial environment, presents formidable obstacles in addressing the repair and regeneration of oral-maxillofacial bone defects. Numerous characteristics should be noticed in oral-maxillofacial bone repair, such as irregular morphology of bone defects, homeostasis between hosts and microorganisms in the oral cavity and complex periodontal structures that facilitate epithelial ingrowth. Therefore, oral-maxillofacial bone repair necessitates restoration materials that adhere to stringent and specific demands. This review starts with exploring these particular requirements by introducing the particular characteristics of oral-maxillofacial bones and then summarizes the classifications of current bone repair materials in respect of composition and structure. Additionally, we discuss the modifications in current bone repair materials including improving mechanical properties, optimizing surface topography and pore structure and adding bioactive components such as elements, compounds, cells and their derivatives. Ultimately, we organize a range of potential optimization strategies and future perspectives for enhancing oral-maxillofacial bone repair materials, including physical environment manipulation, oral microbial homeostasis modulation, osteo-immune regulation, smart stimuli-responsive strategies and multifaceted approach for poly-pathic treatment, in the hope of providing some insights for researchers in this field. In summary, this review analyzes the complex demands of oral-maxillofacial bone repair, especially for periodontal and alveolar bone, concludes multifaceted strategies for corresponding biomaterials and aims to inspire future research in the pursuit of more effective treatment outcomes.

## Graphical Abstract

**Figure:**
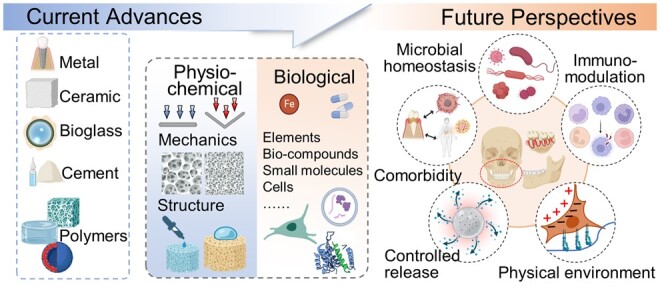

## Introduction

**Figure 1.**
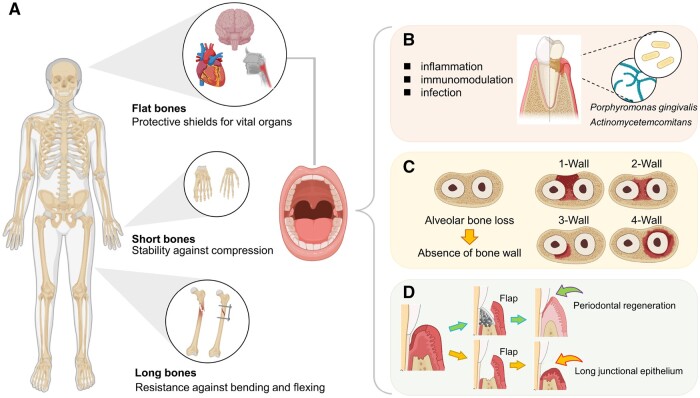
Compared to bones of the trunk, oral-maxillofacial bones exhibit unique characteristics in developmental, anatomical and physiological aspects. (**A**) Identified as a flat bone. (**B**) Vulnerable to bacterial exposure and infection. (**C**) Multi-walled bone defects following periodontal damage. (**D**) Bone regeneration may be interrupted by epithelial attachment. Created with BioRender.com.

The oral-maxillofacial bones, which include irregular bones such as the upper and lower jawbones and alveolar bone, undergo a process of regeneration that shares similar regulatory mechanisms with systemic skeletal bone. This regenerative process can be divided into inflammatory, reparative and remodeling stages, during which various cell populations interact with bone-specific cells to complete the formation of new bone tissue. According to these mechanisms, previous research has extensively investigated various systems of bone repair materials. However, oral-maxillofacial bones possess unique developmental, anatomical and physiological characteristics (Figure 1). Therefore, our objective is to gain a better understanding of the oral-maxillofacial bone in order to facilitate the design of customized biomaterials.

There are several characteristics to consider. Firstly, long bones provide resistance against bending and flexing, while short bones offer stability against compression, and flat bones serve as protective shields for vital organs [1]. As a result, certain regions, such as the edges of alveolar bone, do not require repair materials with high mechanical strength to bear coherent mechanical stress. Secondly, considering the oral cavity’s exposure to bacteria and susceptibility to infection, inflammation and immunomodulation, it is important to pay attention to anti-infective and anti-inflammatory measures and immunomodulatory intervention strategies [2]. Alveolar bone requires enhanced properties against these challenges. Biomaterials that regulate inflammation were employed to preserve the integrity of tooth-supporting alveolar bone [3, 4]. Thirdly, due to the presence of teeth, one-wall, two-wall, three-wall and four-wall intra-bony defects are formed after bone defects in the periodontal structure. Enhancing bone mass without supports from the surrounding periodontal tissues, presents a great challenge [5–8]. Hence, bone regeneration materials used in periodontal area are held to have high osteogenic activity standards.

Moreover, periodontal tissue is a complex structure comprising dental bone, alveolar bone, periodontal ligament and gingiva, making it considerably more intricate than systemic skeletal bone tissue when restoring the anatomical integrity of the entire periodontal structure [9]. During natural periodontal repair, epithelial tissue proliferates rapidly on the surface of tooth root, leading to the formation of long junctional epithelium and deep periodontal pockets [10, 11]. The prevention of epithelial tissue ingrowth largely depends on a blood clot forming between the periodontal flap and the tooth root surface. To address this, oral therapy often includes surgeries like guided tissue regeneration (GTR) or guided bone regeneration (GBR) [11], imposing further criteria for the materials applied. GTR is a periodontal therapy that places a barrier membrane between the bone and the soft tissue, thus to promote the growth of both new alveolar bone and periodontal tissues in areas with bone loss. GBR is an implantology and periodontal therapy that, similar to GTR, utilizes barrier membranes to cover bone defects and prevent fibrous tissue invasion, fostering an environment conducive to osteoblast proliferation and bone regeneration. Clinicians frequently combine membrane and grafting methods, thus membranes becoming an important category among bone regeneration materials [12].

This review aims to discuss the current materials and provide insights into potential advancements in oral-maxillofacial bone repair materials.

## Classification of current bone repair materials

### Classification by composition

#### Metal

Biomedical metal implant provides reliable structural and mechanical support. It addresses the limitations associated with the low mechanical properties of polymer-based materials and the brittleness of bio-ceramics. Titanium (Ti) and its alloys can achieve osseointegration with bone tissues, creating a direct structural and functional link between living bone and a load-bearing implant’s surface, establishing them as the predominant metal materials in biomedical applications [13], thus its application in dental implants is widespread in oral surgery [14]. Additionally, titanium mesh is applied in GTR/GBR across multiple clinical scenarios, and titanium plates and screws are extensively used for repairing significant maxillofacial bone deficiencies and fractures [15]. Additionally, metals such as magnesium, zinc, gold, silver, platinum and their alloys possess unique characteristics such as biodegradability, antibacterial activity and stem cell inductivity [16–18].

Metal materials also have certain limitations, such as implant loosening, infections, lack of biodegradability and a higher elastic modulus compared to cortical bone, which can lead to stress shielding or bone mass loss [19, 20]. To address the lack of biodegradability, biodegradable alloys have become a focus of research in the field. These alloys reserve remarkable mechanical strength, making them suitable for bone defect repair even in load-bearing regions, while the degradation process of these materials not only creates space for bone growth but also stimulates the activity of osteogenic cells through the release of essential ions such as calcium, phosphorus, silicon and zinc [21]. Certain biodegradable alloys, such as Zn-Sr alloys, have demonstrated enhanced properties, with appropriate degradation rates, aperture size and porosity, leading to substantial levels of bone ingrowth [19].

Overall, metal is a traditional material for oral-maxillofacial restorations. To provide better mechanical properties and adapt to dynamic physical microenvironment, further improvements of degradable metals are needed. Additionally, it is also crucial to enhance the antibacterial property and osteo-immune capability of metal implants in oral environment through surface modification such as nanotechnology or bioactive coatings.

#### Inorganic nonmetals

Ceramics serve diverse biomedical applications from dental prosthetics to bone regeneration, while bioglass stands out for its biocompatibility and therapeutic delivery in orthopedic and dental-maxillofacial uses. Based on host-tissue reactions, ceramics are classified into bioinert, bioresorbable and bioactive categories [22]. Bioinert ceramics, such as zirconia and alumina, are typically employed in fixed and removable implant-supported dental prostheses [23, 24]. Bioresorbable ceramics, such as tricalcium phosphates, are often utilized in bone defect repair, particularly in drug delivery and joint replacement, due to their degradable nature that facilitates progressive drug release and space for tissue regeneration [24, 25]. Bioactive ceramics, such as glass ceramics and hydroxyapatite (HA) bioactive glass, are widely used as bone repair material due to their bioactivity, biodegradability and mechanical properties of it surpass those of metals, polymers and bioinert ceramics [26]. The bioactivity of ceramics stems from their ability to mimic the mineral phase of bone, comprising mainly calcium HA, alpha- or beta-tricalcium phosphate, biphasic calcium phosphates (BCPs), unsintered apatites or calcium-deficient apatites (CDAs) [27]; these compositions closely resemble those of the bone mineral phase. Although ceramics possess higher mechanical strength compared to cortical bone, they often exhibit lower toughness and a higher Young’s modulus, leading to limited application for load-bearing bone repair [28].

Compared to ceramics, which possess an ordered crystalline structure, bioglass is an amorphous glass material lacking a distinct crystal arrangement. The primary constituents of bioglass include silica, calcium oxide, phosphorus oxide, sodium oxide, potassium oxide [29], etc. Bioglass offers excellent biocompatibility, bioactivity and osseointegration [30] and efficacy in loading therapeutic molecules and subsequent delivery [31]. Consequently, it has widespread applications in orthopedics as well as cranial-facial and dental-maxillofacial areas [32]. Notably, global regulatory agencies have approved at least 25 classes of bioglass for clinical use [33]. Taking Bioglass (commercial name: 45S5 BG) as an illustrative example, it can be skillfully fabricated into diverse shapes of medical devices and implants, including artificial bones, bone bonders, dental fillings and artificial joints. These exceptional products exhibit remarkable biocompatibility without eliciting any rejection response while effectively integrating with the surrounding tissues to facilitate bone tissue regeneration and repair [34]. Nevertheless, bioactive glasses demonstrate superior regenerative properties compared to other bioactive ceramics but have yet to achieve comparable levels of commercial success [35].

To better adapt to the needs of the oral-maxillofacial system, the following aspects deserve attention: (i) Develop specialized micro-nanostructures to facilitate biological activity. (ii) Enhance mechanical properties such as compressive and flexural strength by synthesizing novel composite biomaterials or developing more sophisticated microstructural designs. Further investigations on scaffold structure and mechanical properties using clinically relevant animal bone-defect models are needed before clinical translation [30]. (iii) Make full use of drug delivery ability, and further construct smart stimuli-responsive system to better meet the complex needs of oral-maxillofacial bone repair.

#### Polymers

Natural and synthetic polymers, each with distinct advantages and limitations, are integral to the development of biomedical materials for bone tissue engineering. Natural polymers, including chitosan, alginate, gelatin, glycosaminoglycan, collagen, fibrin and hyaluronic acid, possess structural and biochemical properties similar to those of the natural bone organic matrix [36, 37]. Some are extensively employed in creating bio-dental materials for oral drug delivery, periodontitis treatment via bone tissue engineering and dentin-pulp regeneration [38]. However, they also exhibit certain undesirable characteristics, such as poor thermal stability, high variability, potential immune rejection, uncontrollable degradation rate, risk of infection and processing difficulty [39].

Synthetic polymers such as poly(lactic-co-glycolic acid) (PLGA), polycaprolactone, polyglycolic acid, polylactic acid (PLA), polyvinyl alcohol, polyethylene glycol, polyurethanes and organic polyols found in some vegetable oils are also prevalent [40]. They are widely utilized as barrier materials in GTR/GBR applications [12]. Moreover, due to advancements in polymeric composites, their prospects in jawbone and maxillofacial applications are also promising [41]. Advantages of synthetic polymers include the stability, cost-effectiveness, low immunogenicity of synthetic polymers and controllable degradation rate. The degradation pace of these polymers can be adjusted to coincide with tissue healing and other limitations can be addressed by surface alteration or mixing with different materials [16]. For instance, PLA, although not structurally ideal for bone tissue regrowth, can be modified to enhance its biocompatibility and mechanical resistance [42]. Nonetheless, there are limitations to consider: (i) Many materials exhibit low hydrophilicity and limited cell adhesion. (ii) While degradation products of synthetic polymers are often metabolizable into carbon dioxide and water or excretable through the kidneys, they can be acidic, potentially inducing sterile inflammation [43]. (iii) Completely removing solvent residues from synthetic scaffolds is challenging; these residues may negatively affect cells and cause inflammation and fibrosis in adjacent tissues [16].

### Classification by structure

Bone repair materials can be classified based on their structures, ranging from macroscopic to nanoscopic scale, such as bulk scaffolds, particles (microscale structures or nanoscale entities) and membranes. They have different functions based on their structures, including stress-bearing capacity, microstructural interaction and biological inductivity.

Scaffolds exhibit superior mechanical properties compared to non-cohesive materials, making them suitable for load-bearing bones such as mandibles and skulls. They are extensively utilized in cranio-maxillofacial repairs for alveolar bone regeneration, mandibular and facial reconstruction and cranial defect restoration. Bone repair scaffold materials can be categorized into metals, inorganic materials, polymers or composites. As an inorganic material, cement consists of powder and liquid parts, demonstrating exceptional plasticity, conductivity, bioactivity and injectability. Calcium phosphate cements (CPCs), including dicalcium phosphate anhydrous and tetracalcium phosphate, are recognized for dental and craniofacial applications [44]. In 1998, CPC became the first FDA-approved injectable bioactive cement [45]. Poly(methyl methacrylate) (PMMA) bone cement is also extensively used [46]. The material’s low-temperature setting reaction and inherent porosity facilitate drug integration. In-depth reviews on cement drug loading strategies have been conducted before [27]. Nonetheless, challenges persist, such as slow biodegradation *in vivo*, porosity dependency on liquid-to-powder ratio and initial powder particle size [27]. As for polymers, it is noteworthy that hydrogel systems excel in maxillofacial bone regeneration with precise involvement of cells and bioactive factors [47, 48]. Hydrogels, formed through physical or chemical cross-linking of 3D polymer networks, exhibit high biocompatibility and mechanical integrity [49], offering notable injectability, degradability, adjustability and straightforward drug loading capabilities [50]. It can be securely fixed within bone defects through bonding or direct chemical connection to bone tissue. However, hydrogels have certain limitations, such as burst release of encapsulated proteins or cells after implantation, inadequate structural stability of natural polymers and unintended bio response.

It is advisable to take advantage of 3D printing technology to shape the overall structure of bulk material. By combining the commonly used oral imaging technique, cone beam computed tomographic (CBCT) with 3D printing, it is possible to individually customize bone grafts that not only precisely fit the irregular bone defects of the oral-maxillofacial region, especially alveolar bone defect, but also with desirable pore structure to load and release various bioactive components [51–55]. Compared to conventional bulk grafts, 3D-printed bone grafts offer a larger contact area due to their close-fit with the bone defect, resulting in greater new bone volume post-repair [56]. However, there are still some practical problems. For instance, there is no standard CT value of CBCT to recognize the actual size and shape of bone defect under the interference of residual granulation, soft or hard callus tissue [56]. Additionally, the defect area is often with complex anatomies such as undercut or sharp corners to hinder the close-fit of the implants. 4D printing material with shape memory could solve these problems. With this method, the original and subsequential shape and size of bulk could be programmed before implant. After implantation, the scaffold would change morphology to close-fit the actual bone defect precisely over time [57]. However, the currently reported 4D printing material used in oral-maxillofacial bone repair mainly focuses on timely controlled release of bioactive components [51–54]. In the future, shape-memory materials for periodontal bone repair need further research [58].

Particle-based materials could fill bone defects with various complex morphological structures, thus find extensive applications for diverse forms of defects such as alveolar bone defects [15]. Currently, the focus of clinical application primarily revolves around artificial bone powder (e.g. Bio-Oss) and artificial bio-ceramic composites, both are composed of calcium-phosphorus compounds. However, the osseointegration effect of Bio-Oss is not optimal, as it cannot be fully absorbed. It is unfavorable for subsequent treatments requiring tooth movement such as orthodontics. Therefore, it is important to focus on improving the absorbability of such materials. Particles can be further categorized into micron-scale (≥1 μm), submicron-scale (100 nm–1 μm) and nanoscale (≤100 nm) dimensions [59]. The design of macro-nanomorphology is a considerable strategy for manufacturing advanced bone replacement scaffolds [42, 50] for they directly regulate protein adsorption, activate osteoblasts and promote osteogenesis, creating an optimal microenvironment for bone regeneration. Additionally, compared to conventional HAp ceramics with low mechanical strength, nanocrystalline HAp powders exhibit improved sinterability and densification due to their larger surface area [60]. Moreover, under some circumstances, micro-sized tricalcium phosphate particles were identified as promoting M2 Macrophage polarization and promoting MSC-based bone formation [61]. It is noteworthy that nano/micromaterials include not only particles but also nanorods, nanoflakes and many other nano/micro scale morphologies.

Membranes are essential to prevent the formation of long junctional epithelium, deep periodontal pockets and restore anatomical structures during periodontal regeneration. These membranes have various applications, including GTR/GBR, alveolar ridge preservation, vertical ridge augmentation and sinus floor augmentation window coverage [62]. Membranes can be classified as either non-degradable barrier membranes or degradable ones. Non-resorbable membranes demonstrate good mechanical properties, enabling them to retain their shape and provide space for new tissue formation [62], thus allowing for precise control over their barrier function [11]. A systematic review concerning vertical bone gain revealed that GTR techniques using non-resorbable membranes yield the most favorable results [63]. However, it is subject to certain limitations. (i) It necessitates a subsequent surgical intervention for its removal, leading to increased morbidity and patient discomfort. (ii) It lacks the ability to seamlessly integrate with the surrounding tissue. (iii) Its brittleness poses another challenge in its application [11, 12]. Resorbable membranes have enticing potential for employing guided tissue regeneration as a one-step procedure. Nonetheless, their insufficient rigidity compromises shape retention compared to non-resorbable membranes, particularly upon exposure to oral fluids or blood [12]. Therefore, it is essential that degradable barrier membranes are designed to resorb in synchrony with periodontal regeneration, ensuring their degradation is not far faster or later than healing [62].

Except for barrier function, in order to meet the physical microenvironment and the antibacterial demand of periodontal regeneration, membranes need to develop multiple functions, such as angiogenic function, osteoinductivity and antibacterial activity. For example, Song *et al.* [64] developed a graded series of flexible BaTiO_3_/P(VDF-TrFE) electroactive nanocomposite membranes with varying surface charge intensities, thus to achieve wide spectrum bactericidal effects and promotes tissue regeneration. As for the angiogenesis function, the physical properties such as porous structure of the membrane are far more important than delivery of various bioactive compounds [65]. Detailed modification strategies are in the “Current modifications of bone repair materials” section.

Promoting periodontal regeneration through the combination of membranes and bone substitutes remains complicated. Compared to optimizing them separately, it is more innovative to inventing an integrated material with pro-regenerative polarity to repair the periodontal structure by a single operation. For example, a patent has explored an integrated material termed as ‘regional function-specific clinical periodontal defect repair module’ [66]. Lei *et al.* configured these bone grafts with distinct domains: alveolar bone regeneration functional domains, periodontal ligament regeneration functional domains and barrier membrane functional domains, thus promoting the regeneration of periodontal ligaments and alveolar bone separately to finely restore periodontal tissue. Unfortunately, beyond this, there is few research on such integrated materials.

## Current modifications of bone repair materials

### Aligning mechanical properties of biomaterials with natural bone

Optimal mechanical properties for bone defect treatment are application-specific. Cortical autografts require high initial strength, whereas cancellous bone autografts, owing to their porosity, do not necessitate such rigidity [12]. If bone repair materials exhibit mechanical properties markedly distinct from adjacent bone tissue, stress shielding and related phenomena may cause damage and fatigue in neighboring tissue and inhibit integration and regenerative capacity, thereby impeding bone regeneration [21]. Different mechanical properties of compact and trabecular bone were summarized in others’ work (Table 1) [40, 76]. Stress–strain curve for cortical bone and compressive stress–strain curves for several relative densities of wet cancellous bone were summarized [77]. Projection-based 3D printing enables the creation of hydrogels with graded density that emulate the porosity of cancellous bone and the hardness of dense bone. This method efficiently balances load distribution and minimizes stress concentration at dental implant sites [78]. Similarly, a multi-domain gel scaffold has been engineered to foster a gradient from chondrogenic to osteogenic traits, simulating the natural gradation between calcified cartilage and the subchondral bone plate, along with the unique biological, topological and mechanical zones of cartilage [79]. These mechanical properties and corresponding repair strategies are suitable for oral-maxillofacial bones with distinct cortical and cancellous structures.

**Table 1.** Main properties of cortical and trabecular bone

	Cortical bone	Trabecular bone	References
Composition	Mineral phase: hydroxyapatite (Ca_10_(PO_4_)_6_(OH)_2_), carbonate, citrate, Na^+^, Mg^2+^, Cl^−^, organic phase: collagen type I, noncollagenous proteins, lipids. Water		[67, 68]
Porosity	5–10%	50–95%	[69]
Pore size	9–100 μm	3.50 × 10^−3^–9.50 × 10^3^ μm	[70, 71]
Young’s modulus	7–30 GPa	0.05–0.5 GPa	[72, 73]
Compressive elastic modulus	11.5–17 GPa	0.12–1.1 GPa	[72]
Tensile strength	50–151 MPa		[72, 74]
Compressive strength	130–200 MPa		[72, 73]
Fracture toughness	2–12 MPa m^1/2^		[72, 75]

Reproduced with permission from Ref.[40], Copyright 2022, Wiley.

The mechanical requirements of periodontal bone repair are not as strict as long bone and flat bone. Bone powder and block-type collagen are already sufficient to meet unstrict mechanical demands, so they are widely used in clinical practice. However, with loose mechanical structure and required months of healing duration, we suspect they are actually not idea bone grafts in some surgeries such as maxillary sinus augmentation or alveolar bone increment surgery required subsequent dental implant. In this scenario, the bone substitute should provide significantly enhanced mechanical properties in time to support implants [80]. Bulk, such as cement or 3D printed bone graft may conduct rapid osseointegration and promptly provide sufficient mechanical supports for implants, thus could be an ideal bone graft material under this scenario. For practical application, an interesting scaffold composed of PLGA, HA and beta-tricalcium phosphate was fabricated by 3D printing technology. It was designed with a pre-tapped hole inside to fit the dental implant, thus to achieve alveolar bone augmentation and stable dental implant placement via one-step surgery. Results of micro-CT and histological analysis confirmed that this scaffold was able to obtain adequate stability quickly [81].

In addition, the mechanical properties during bone regeneration are dynamic. Certain bone defects, such as those resulting from tooth extraction, undergo tissue remodeling facilitated by machine-based techniques following clot formation. This process gradually enhances the mechanical strength of the affected site. Certain materials exhibit a dynamic hardening ability to accommodate this transformation, thereby ensuring consistent mechanical properties throughout the entire procedure, such as Yu’s work mentioned below [82]. It is also reported that a 3D-printed elastomeric nanohybrid scaffolds with thermo-responsive mechanical properties could be softened by reverse self-assembling at a certain body temperature, thus relaxing stiffness and favoring osteogenic activity [83].

### Optimizing surface topography and pore design enhances wettability, biomimicry, adhesion and osteoimmunomodulation

Surface topography and pore architecture are two key factors dominant the physiological microenvironment and mechanical characteristics [84], impacting cellular activity and modulating local microenvironments at molecular and cellular scales to induce cell adhesion, spreading, proliferation and differentiation [59].

**Figure 2.**
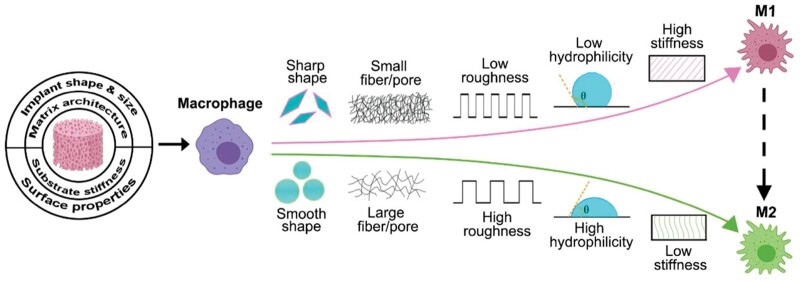
The structure of materials, including surface roughness, hydrophilicity and hardness, regulates the macrophage response. Reproduced with permission from Ref. [87], Copyright 2021, Wiley.

Surface topography critically affects surface area, roughness, wettability, charge and robustness, and promotes a conducive tissue integration and immune environment [21, 42, 59, 85, 86], such as modulating the polarization of macrophage (Figure 2). For instance, micro to nanoscale modifications ranging from 1 up to 100 nm on titanium influence hydrophilicity, mechanical integrity, integration and antibacterial properties [88]. Concurrently, metal components can be incorporated into other scaffolds or utilized as nanoscale surface coatings, owing to their remarkable bioactivities. For example, metal-organic framework (MOF), a synthetic material consisting of coordinated metal species and organic ligands, has shown potential in modulating the bio-implant interface to enhance implant osseointegration [89]. Additionally, metal-based nanocomposite biomaterials, such as nano zirconia, silver nanoparticles, etc., are widely used in bone tissue regeneration [90], for nanoscale morphology provide favorable optimal cues and microenvironments for cell growth and differentiation to the material [91]. Similarly, using Mg-enriched HA or controlled nanotopography on ceramics benefits bone growth and stem cell behavior, thus enhancing implant success and longevity [13, 88, 92, 93]. On a more microscopic level, natural polymers are frequently utilized as coatings due to their good affinity for cells. The presence of hydroxyl, carboxyl, amino, aldehyde groups or functional peptides in these natural polymers can significantly improve osteoblast-material adhesion via protein interactions or calcium chelation [86]. Surface functionalization with -NH_2_ and -COOH groups on scaffolds also facilitates stem cell attachment and osteogenesis by forming hydrogen bonds with fibrinogen [94].

Currently, to accelerate the osseointegration process of dental implants, the surfaces are usually roughed up by sandblasting and acid-etching [95]. However, rough surfaces exposed to the oral cavity are vulnerable to plaque adherence and bacterial colonization, thus lead to infections such as peri-implant diseases, with prevalence rate of 43% across Europe [96]. The implant surface can modulate the biofilm and disease-associated microbiome [97, 98]. For example, the electrostatic forces and ionic bonds that lead to initial bacterial adhesion are quite different between implant alloys and HA [97]. As a result, the primary preventive strategy of peri-implantitis is surface modification which could inhibit biofilm formation. Yu *et al.* [99] summarized these modifications, such as introducing functional coatings or antibacterial surface topography. Nevertheless, these strategies with positive outcomes are rarely transformed into clinical application [99]. One of the reasons is that, the actual microbial composition and organization of periodontal flora are much more complicated than that of *in vitro* sterile environment or *in vivo* experimental animals lived in SPF barrier environment. In this case, we recommend an *in vitro* 3D anatomical gingival tissue model [100], which is fabricated from silk biopolymer and effectively simulates the structural, physical and metabolic conditions of human oral gingival tissue, such as the native oxygen gradient within the gingival pocket. This model could provide the human subgingival plaque microbiome. We could further develop and apply it to investigate disease-associated microbiomes in peri-implantitis and antibacterial surface optimization. Furthermore, surface modification could also focus on immunoregulation in periodontal tissues. Some researches indicate that the impact of surface roughness on peri-implantitis risk is rather limited [101, 102], whereas local immune dysregulation induced by periodontal disease and smoking is the main cause [103]. Chen’s [85] work provides a valuable reference in surface immunoregulation, such as hydrophobic and cationic materials are more likely to induce immune response.

The pore structure includes pore size, porosity and the interconnectivity and penetration of pores, etc. While high porosity may compromise the mechanical strength of bone scaffolds [104], optimal regeneration outcomes require a porosity exceeding 90% [40]. Porosity and pore size serve as indicators for mechanical strength, cell settlement and migration [59]. Discussions around pore size range from 1 to 1000 μm across different scaffold designs. Magnesium phosphate scaffolds featuring micropores below 25 um exhibit superior lamellar structures, facilitating rapid biodegradation and enhanced calcification [105]. Uniform pore sizes between 30 and 40 um promote polarization of RAW cells towards M2 type while imperforate or randomly distributed pore sizes tend to polarize RAW cells towards M1 type [85]. Also, there have been studies suggesting that the optimal pore size should range from 50 to 150 μm [106]. Pores measuring between 90 and 120 μm have been found to impede angiogenesis and promote chondrogenesis, whereas larger pores (350 μm) facilitate angiogenesis, resulting in increased oxygen tension and nutrient supply [107]. Some researchers argue that bioglass materials with pores ranging from 500 to 850 μm exhibit the highest reactivity towards osteogenesis [108]. There is also implant with pore sizes larger than 1000 μm [106]. To conclude, larger pores in bone scaffolds facilitate blood vessel growth and relative hyperoxia, thus enhance vascularization and mineralization; smaller pores improve bioactive molecule adsorption, aiding in nutrient distribution [85]. Appropriately small pore induce moderate hypoxia, promoting angiogenesis and bone repair, whereas excessive hypoxia may lead to inflammation and barriers to bone integration [109]. Most studies to date, despite the absence of standardization, suggest pores ranging from 350 to 500 μm to encourage vascularization and new bone growth [40, 110, 111].

There is no specific research on the pore structure for alveolar bone substitutes. The microbial distribution within gingival pockets exhibits clustering patterns [112]. Aerobic and facultative anaerobic bacteria are predominantly found on the enamel or cementum at the gingival margin, whereas obligate anaerobic bacteria, which play significant roles in oral diseases [113], are more prevalent near the gingival pocket bottom. This distribution is highly correlated with the oxygen gradient within the gingival pocket [100]. As previously mentioned, pore structure will influence the oxygen permeability of materials, and even could regulate hypoxic or relatively hyperoxic microenvironment. Manipulating material structures to adjust oxygen levels, and thus influence the colonizing bacterial flora could be a promising therapeutic approach worthy of investigation.

The interconnectivity and penetration of pores replicate cancellous or cortical bone structures, providing cues to cells that mimic components of the extracellular matrix (ECM) [21, 84]. For example, using digital light processing technology to create integrated bilayer scaffolds with ‘lotus- and radial-’ distributed pores has shown to guide chondrocytes and mesenchymal stem cells effectively to defect sites, demonstrating significant osteochondral repair in rabbits [114].

### Modification by adding bioactive component for enhanced performance

To enhance biological properties of bone repair materials, bioactive components can be integrated. These components range from elements to drugs, biological compounds and cells. They can stimulate osteogenesis-related cell lines, augment progenitor cell migration and differentiation, and encourage osteoblast adhesion, migration and proliferation. Additionally, they modulate bone immunity, provide immunoregulation and target angiogenic cells and cytokines to boost vascularization. Furthermore, they support nerve regeneration by influencing the periosteum.

#### Bioactive elements

**Figure 3.**
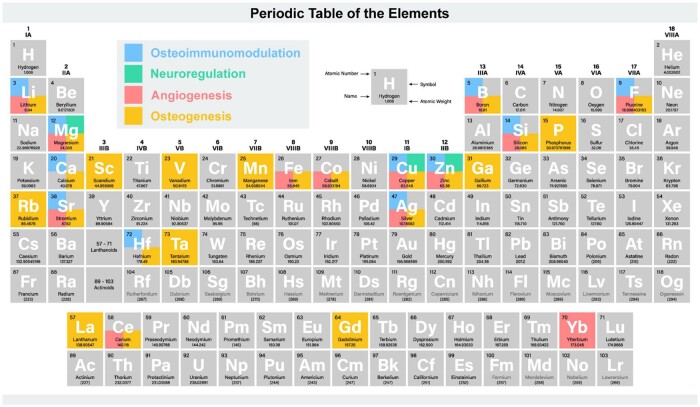
The role of bioactive elements in bone regeneration. Reproduced with permission from Ref. [122], Copyright 2023, Open Access.

Beyond basic bone components, certain rare metal elements are often explored for their bone regeneration potential. These ions play diverse roles, including catalyzing protein folding, facilitation of cellular uptake and receptor interactions. These activities enable osteoimmunomodulation, neuroregulation, angiogenesis and osteogenesis [115]. Elements like Se, Zn, Mg and Ta are known to significantly enhance mineralization [86]. For instance, Mg ions influence the expression of bone-related genes, including Runx2, ALP, OCN and OPN, through the phosphatidylinositol 3-kinase (PI3K)/protein kinase B (Akt) signaling pathway [116]. Ca, Zn, Sr, Mg, B, Ti, Cu and phosphate anions are also crucial for angiogenesis and vascularization [115]. For example, in Xu’s work, bone marrow stromal cells (BMSCs) convert L-arginine into nitric oxide (NO) in the presence of Ca^2+^, activating the NO/cyclic guanosine monophosphate (cGMP) signaling pathway. Concurrently, NO, along with Ca^2+^ and L-arginine, acts as external enhancers, promoting osteogenesis-angiogenesis coupling in surrounding BMSCs and endothelial cells at the bone defect site [117]. Ca, Mg, Zn, Sr and Co modulate immune responses and influence osteoimmunoregulation [85, 118] through pathways such as PI3K/Akt, glucose transporter 1, autophagy and nuclear factor-κB (NF-κB) [119, 120]. Co and Si may increase inflammation, whereas Zn, Mg and Sr can decrease it [85]. Some elements possess multiple functionalities. For instance, Sr can promote osteogenesis, stimulate angiogenesis and reduce inflammation in bone grafts [121]. Bai *et al.* [122] provided a comprehensive summary of the roles of bioactive elements (Figure 3) and in-depth descriptions of the mechanisms.

However, extensive research indicates that the mechanisms of elements’ osteogenic effects are not yet fully understood. Generally, an element possesses multiple functions. For instance, Co ions are pro-inflammatory, they also have been used to accelerate vascularization due to their capacity to stabilize HIF-1 and activate vascular endothelial growth factor (VEGF) [123]. The mechanism between these multiple functions in the osteogenic process is not clear. Additionally, there are many valence states of an element. Different valence of the same element may exert different functions. For instance, silver nanoparticles can promote osteogenic lineage induction and actin aggregation in new bone formation, whereas silver nitrate (AgNO_3_) cannot [122]. The exact mechanisms need to be further investigated.

#### Macromolecular bio-compounds

During different phases of endogenous bone repair, cytokines and signaling molecules are released sequentially to regulate osteogenesis [21]. Materials aiding this process should mimic natural healing by providing physiological release profiles and spatial gradients [124]. List of products associating bioactive molecules (growth factors [GFs], peptides or small molecules) with a material carrier and their stage of development has been documented previously [21].

Cytokines including interleukins, interferons, colony-stimulating factors, tumor necrosis factors and GFs are key in regulating anti-oxidative, anti-inflammatory and osteogenic activities. Among these, GFs are particularly used to enhance cellular proliferation with various functions [124]. Key GFs include transforming GF-β, epidermal growth factor, VEGF, fibroblastGF, nerveGF, platelet-derived GF and others. Platelet lysate, like platelet-rich plasma (PRP), which is rich in GFs, has been shown to improve bone healing [12]. A single GF could be multiple functions, such as Nel-likemolecule-1, as a secreted osteoinductive protein, controls the amount of bone formed, promotes cartilage regeneration, induces pericyte proliferation and has pro-angiogenic effects [21].

Regarding peptide hormones, parathyroid hormone (PTH), the only FDA-approved anabolic treatment for osteoporosis [125], is commonly utilized as a pharmaceutical agent for the treatment of osteoporosis [21]. Low calcium levels induce PTH production, which subsequently activates the Wnt-beta catenin pathway crucial for bone formation. Additionally, peptides like glucose-binding proteins and Gox/CAT can be integrated into smart biomaterials that respond to environmental cues, enabling controlled release mechanisms [126]. Although these peptides may do not directly promote osteogenesis, they play a crucial role in regulating material degradation and controlling drug release within the structural system [127].

The functions and applications of proteins include bone morphogenetic protein (BMP), Enamel matrix derivative (EMD), peptide hormones and others. Among them, BMP-2 and BMP-7 have been extensively studied and are considered the most mature in terms of research and application. Notably, rhBMP-2 is currently the sole bone-induced bone graft that has undergone rigorous testing and received approval from the FDA [12]. It has been postulated that these proteins primarily composed of amelogenin may potentially stimulate cementum deposition as well as periodontal regeneration [128].

#### Osteogenic small molecules

Osteogenic small molecule is also one of the encouraging approaches to regenerate bone tissue. Compared to other bio-compounds derived from living organisms, such as GFs, platelet lysates, hormones and plant hormones, abiogenic drugs exhibit a simpler molecular structure, enhanced physio-chemical stability and a more direct mechanism of action. Pathways governing bone mass can be selectively targeted using small molecules [21]. Various organic and synthetic compounds can promote bone healing by enhancing mineralization, collagen synthesis, and cell signaling and differentiation [129]. These include flavonoids, curcumin, vitamins and common medications such as aspirin, ibuprofen and EDTA. Small-molecule amines such as melatonin and metformin also support bone maintenance and regeneration, with many already approved by the FDA for clinical use [130]. This indicates the potential for developing biomaterials incorporating such drugs and molecules [131].

However, the challenges posed by small molecules include poor water solubility, rapid degradation and non-specificity, often causing therapeutic failures [132]. Thus, advanced delivery systems ensuring precise, controlled release of hydrophobic and neutral compounds are critical. Techniques such as micro/nanocapsulation, physical adsorption or conjugation are potential strategies [133]. Osteoinductive agents, antibiotics, alendronate, simvastatin, raloxifene and immunomodulators, including steroids and NSAIDs, have been integrated into such delivery matrices [85, 134, 135].

Although there were extensive researches on the mechanism of osteogenesis-related bioactive components, their practical applications in research usually were not rigorous. For instance, suppressing inflammation from the beginning may not favor wound healing. Still, many researchers try to exert anti-inflammation effect of these bioactive components in the early stage, while ignoring the necessity of early inflammation. For another example, the transition of macrophages from the M1 to M2 phenotype generally represents the shift from pro-inflammation to tissue regeneration. Therefore, studies conducted immunomodulatory therapies based merely on promoting M2 polarization. In recent years, researchers found that M2 macrophages can be divided into multiple subtypes from M2a to M2f with diverse inducers and selected functional properties during bone regeneration [136]. For example, M2a and M2b exert immunoregulatory functions, whereas M2c macrophages are more related to the suppression of immune responses and tissue remodeling [137]. Consequently, bioactive components targeting macrophages should avoid an all or nothing strategy [136], while focusing on subtypes regarding specific functions.

Ideally, bioactive components should target periodontal-specific microenvironment. For example, Zhu and his team found that quercetin specifically targets orofacial mesenchymal stem cells (OMSCs), reducing oxidative stress and influencing osteogenic growth via molecular mechanisms that regulate m6A modifications in Per1. So, they combined quercetin with a specially designed material which could adapt to the dynamic and moist periodontal environment [138]. For another example, Zhang’s team conducted systematic studies on CD301b+ macrophages closely associated with periodontitis bone loss [139, 140], and designed an osteogenic inducible nano-capsule (with a gold nanocage containing IL-4 serves as the ‘core’, and the membrane of neutrophils serves as the ‘shell’) to target CD301b+ macrophages. To conclude, researchers need to profoundly understand the underlying biomolecular mechanisms of periodontal diseases to better applying these bioactive components.

### Loading cells and their derivatives to endow bioactivity in biomaterials

Cell transplantation for bone repair was first documented over five decades ago [141], yet few treatments have reached clinical practice. While direct cell delivery in biomaterials may provoke immune reactions, using autologous or modified cells shows therapeutic promise. T cell delivery, especially in cancer immunotherapy, is a key research area, where patient-derived immune cells are activated or engineered for tumor specificity, then reinfused after expansion. T cells encapsulated in biomaterials aim for a targeted immune attack on tumors. However, similar approaches for bone regeneration are limited, likely due to ethical and logistical issues [124]. It is worth to mention that exosome therapies from immune cells offer a more feasible route, promoting osteogenesis and aiding fracture repair [142].

Natural bone comprises four distinct cell types embedded within the ECM: osteoblasts, osteoclasts, osteocytes and bone lining cells [42]. However, a broad spectrum of cell types is available for incorporation into biomaterials for bone repair. Commonly, BMSCs, adipose-derived mesenchymal stem cells (ASCs) and periosteum-derived stem cells (PDSCs) are used, with ASCs being readily harvestable and PDSCs noted for their osteogenic differentiation capabilities [21]. Six different types of dental stem cells (DSCs) have been identified and studied in detail [143], they can repair bone, dental and soft tissue, and also treat inflammatory diseases such as myocardial infarction, colitis, wound healing and type 2 diabetes. For maxillofacial bone regeneration, a variety of cells, especially for periodontal use, are summarized in Table 2. Amanda and colleagues [164] have thoroughly reviewed cell delivery strategies.

**Table 2.** Cells used for maxillofacial bone regeneration

Cell type	Advantage	Disadvantage	Application
Bone marrow stem cells (BMSCs)	Well self-renewal, migration and pluripotency [144]. Proficient immunosuppressive properties [145]. Strong osteogenic differentiation potentials [146].	Lower isolation success and proliferation rate compared to adipose tissue mesenchymal stromal cells [145, 146].	The most used stem cells in cell therapy and tissue repair [144]. Alveolar bone tissue engineering [147].
Adipose-derived stem cells (ASCs)	Most advantageous to isolate [148]. Rapid proliferation [145]. Long periods of differentiation capacity [149]. Immunosuppressive effect [145]. Immunomodulatory properties [150].	Unbefitting for endochondral fracture healing [21]. Factors affecting ASC differentiation are relatively unknown.	Stabilized fractures with intramembranous bone healing [21].
Periodontal ligament stem cells (PDLSCs)	Rapid proliferation. Multi-lineage differentiation potential. Fine osteogenic differentiation potential. Strong self-renewal ability [151].	Limited number of stem cells. Stem cell damage caused by donor quality. Risk of tumor [152].	One of the most promising cells in periodontal regeneration therapy [151].
Stem cells from human exfoliated deciduous teeth (SHEDs)	Rapid proliferation and differentiation [153]. Better osteogenic ability than DPSC and BMSC [154]. Promising capacity in regenerative medicine [155]. Fewer ethical concerns. Low immunogenicity and tumorigenicity [156].	Further research is needed to optimize the use of SHED-derived secretome [156].	SHED-derived secretome [156]. Preclinical models of neurodegenerative diseases [157]. Cartilage regeneration [158]. Dental pulp regeneration [159].
Dental pulp stem cells (DPSCs)	Strong multi-lineage differentiation capacity. Plastic adherent ability. Immunomodulatory features [155]. The same tissue origin as craniomaxillofacial bone and periodontal tissue (ectodermal mesenchyme derived from cranial nerve ridge) [160]. Well chondrogenic differentiation ability [21].	Decreased proliferation and differentiation rate. Elevated senescence and apoptosis with increasing age.	Endodontic regeneration. Pulp-dentin complex regeneration. Extraoral tissue repair and regeneration [161].
Body fluid-derived stem cells (BFSCs)	Stemness properties. Multi-differentiation potential. Immunomodulatory effects [162].	Unknown efficacy and safety before therapeutic translation [162].	Repair of genitourinary abnormality [162]. Amniotic fluid-derived stem cells combined with PRP show bone regeneration ability [163].

**Figure 4.**
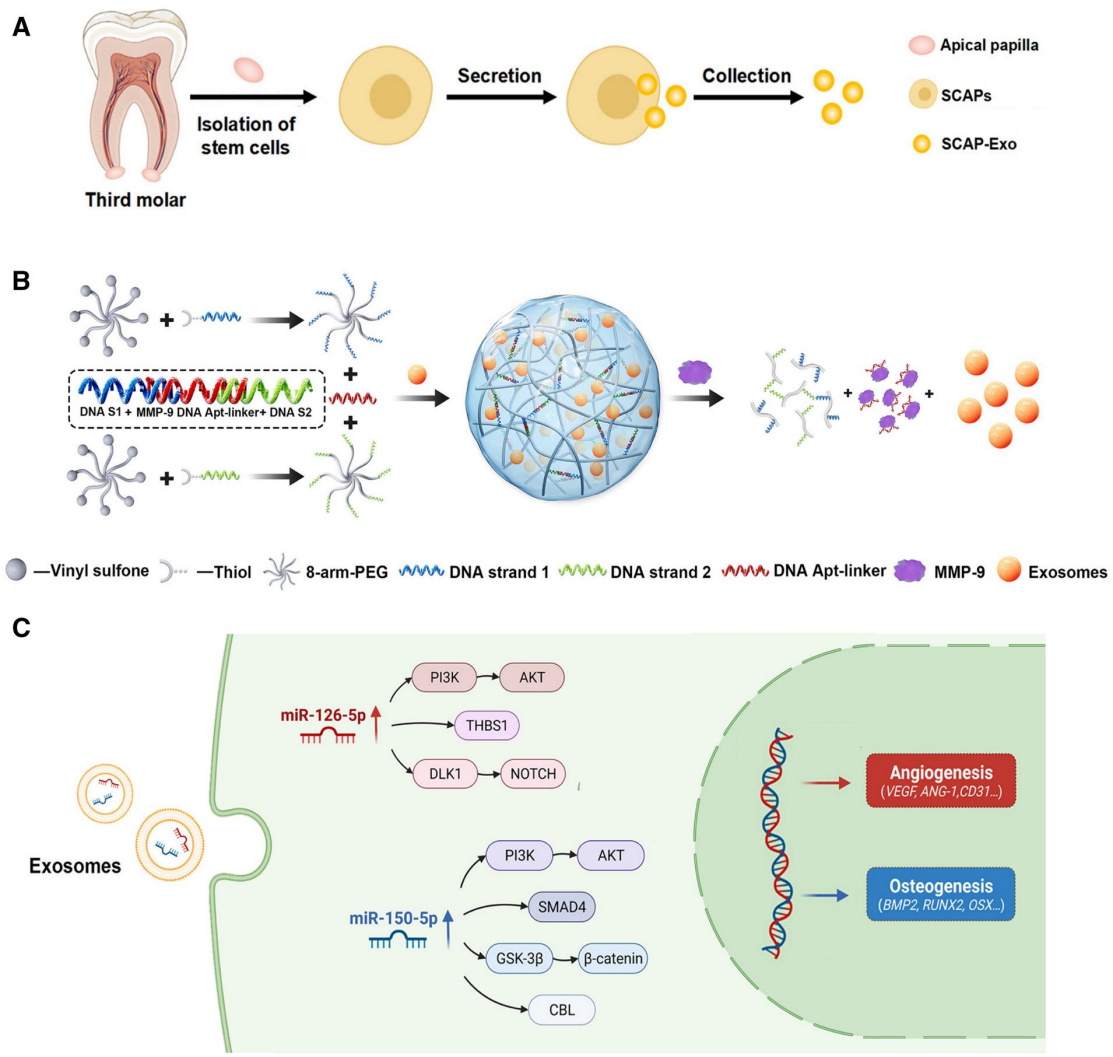
A bio-responsive DNA hydrogel loaded with stem cells from apical papilla-derived exosomes (SCAP-Exo) enhances bone regeneration in diabetes. (**A**) SCAPs isolation and SCAP-Exo extraction. (**B**) The synthesis and responsive behavior of SCAP-Exo-loaded PEG/DNA hybrid hydrogel. The system activates in response to diabetic conditions via matrix MMP-9, releasing exosomes that enhance angiogenesis and osteogenesis. (**C**) Potential signaling pathways mediating angiogenesis and osteogenesis enhancement by miR-126-5p and miR-150-5p from SCAP-Exo. Reproduced with permission from Ref. [168], Copyright 2022, American Chemical Society.

Exosomes are nanosized vesicles with a lipid bilayer capable of carrying proteins, RNAs and other molecules, and they exhibit high targeting efficiency [165]. MSC-derived exosomes can improve cell migration, osteogenesis and angiogenesis through various signaling cascades, including BMP/Smad, Wnt/β-catenin and PI3K/AKT pathways [166, 167]. They can be incorporated into biomaterial scaffolds for targeted delivery to injury sites, and offer advanced gene editing potential when compared to alternative methods [168]. For instance, exosomes engineered with the VEGF gene, combined with 3D-printed scaffolds, can enhance osteogenic differentiation and regulate vascular remodeling [169]. Furthermore, bio-responsive exosome systems have been developed for mandibular bone regeneration in diabetic conditions, utilizing a PEG/DNA hydrogel that releases exosomes with angiogenesis and osteogenesis function in response to the elevated pathological cue like metalloproteinases-9 (MMP-9) (Figure 4) [168]. Given that the mechanical perception and response of periodontal soft tissue, specifically periodontal membrane fibers, play important roles in alveolar bone remodeling, more concerns should be taken on periodontium-origin exosomes. A recent study showed that healthy PDLSCs-exosomes suppressed the over-activation of Wnt signaling to recover the osteogenic differentiation capacity of inflammatory PDLSCs, and they successfully accelerated bone formation of alveolar bone defect with periodontitis [170]. Meanwhile, in orthodontic treatment, tension stimulation leads to alveolar bone regeneration while pressure results in bone resorption, where PDLSCs that sensitive to stress and tension play a pivotal role. Based on the phenomenon, Wang *et al.* [171] found exosomes secreted from the PDLSCs stimulated with tension significantly enhanced the osteogenesis of BMSCs compared with those from untreated PDLSCs. They further clarified that mechano-responsive micro-RNA miR-200b/c carried by these exosomes targets Smurf1 to activate the BMP-Smad signaling pathway, thus promoting osteogenic differentiation of mandibular BMSCs [172]. Their works suggest a novel therapeutic for bone loss in periodontitis.

Cell-based gene therapy uses genetically modified cells, especially stem cells, as vehicles for therapeutic genes to enhance their osteogenic differentiation through the secretion of osteogenic factors [124]. Research has shown that modifying the epigenome of osteoblasts can accelerate mineralization and enhance the osteoinductive properties of secreted extracellular vesicles [173]. This approach has been used in gene therapy for bone regeneration and even the regrowth of teeth, roots and periodontium [174]. Another method of gene delivery involves directly introducing therapeutic genes into the body using viral or non-viral vectors, RNA-mediated vectors or genome engineering techniques such as CRISPR/Cas9 [124, 175–179]. For example, drug delivery systems for CRISPR-based genome editors can precisely manipulate genetic targets [180]. While the majority of non-viral delivery approaches for CRISPR/Cas9 rely on micro-/nano-particle delivery techniques, it is possible to immobilize Cas9: sgRNA lipofectamine complexes onto biomimetic fiber scaffolds [181]. This method enables localized, sustained and non-viral delivery of Cas9: sgRNA complexes, ultimately facilitating efficient gene editing in cells seeded on the scaffolds.

## Future perspectives for enhancing oral-maxillofacial repair materials

### Manipulating the physical microenvironment for enhanced healing

Physical factors, both static and dynamic, significantly influence bone repair. Changes in physical factors may be static or dynamic, such as physiological potential and progressive stiffening during the process of the blood clot to the blood callus and then to the callus. Oral-maxillofacial bone, particularly alveolar bone, has the highest regeneration rate among all bones in the body [182]. Tissue repair materials need to adapt to these changes in natural physical factors. However, current materials lack sufficient dynamic mechanical and piezoelectric properties to fully take part in cellular mechano-transduction and also lack technologies to react the complex interactions between various physical cues [183].

**Figure 5.**
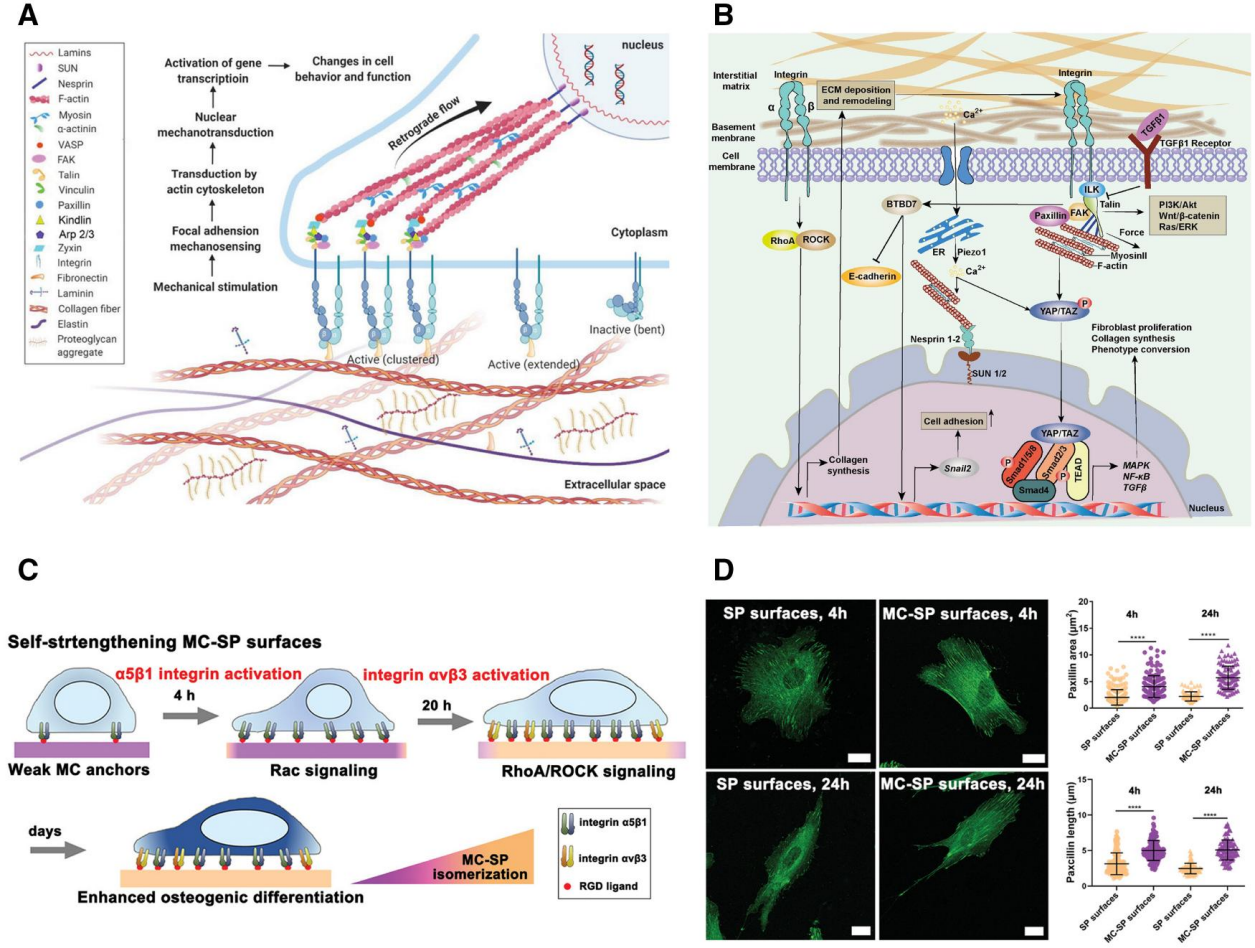
Cellular mechano-transduction and its application. (**A**) Cellular mechanosensing process includes mechanical stimulation, force transmission through actin filament—protein chains, mechanical signaling conversion and activation of transcriptional factors and transcripts. Reproduced with permission from Ref. [183], Copyright 2023, Wiley. (**B**) The key molecular mechanism of cellular mechano-transduction. Reproduced with permission from Ref. [184], Copyright 2023, Open Access. (**C**) Schematic view of the adhesion, differentiation and related signaling pathways for stem cells on the self-stiffening MC–SP coating and SP surfaces. (**D**) The area and length of focal adhesions, as indicated by the distribution of paxillin, were greater on the self-strengthening MC–SP surfaces compared to the static SP surface either in early (4 h) or matured (24 h) adhesion stages. The scale bar indicates 20 μm. Reproduced with permission from Ref. [82], Copyright 2020, Wiley.

Mechanobiology widely affects bone homeostasis [184]. At the macro level, mechanical loading of different magnitudes, frequencies and types can regulate the biological functions of bone tissue through the mechano-sensor of bone cells [185]. It was proved that mechanical loading applied to the cell-scaffold composite could dually modulate the inflammatory responses and osteogenic differentiation of MSCs [186]. On the micro level, cells could communicate via the intracellular skeleton and cell surface adhesion molecules with the ECM, such as the curved nanofibers in the ECM microenvironment, thus regulating osteoclast differentiation and contributing to bone homeostasis [187, 188]. The conversion of mechanical signals into biochemical responses within cells is known as cellular mechano-transduction [184]. This procedure involves the application of mechanical stimulation, the transmission of force through actin filament-protein chains, the conversion of mechanical signaling and the activation of transcriptional factors and transcripts (Figure 5A) [183]. The key effectors responding to mechanical stimulations include Piezo channels, integrins, Yes-associated protein (YAP)/transcriptional coactivator with PDZ-binding motif (TAZ), transient receptor potential vanilloid 4 (TRPV4), etc. (Figure 5B) [184]. Ultimately, this process directs the cellular functions and phenotypes. Besides, mechanical cues in the communication loop between ECM, non-hematopoietic cells and immune cells play a role in local inflammation [189]. Yu *et al.* [82] proposed biomaterials, with a self-strengthening polymer coating that mimics the dynamic nature of the native ECM, can spatiotemporally impart biophysical cues to manipulate cell fate. Spiropyran (SP) serves as a dynamic anchoring group to control the binding strength of cell adhesive peptide ligands. Benefiting from the spontaneous thermal isomerization from merocyanine to spiropyran (MC–SP), the self-responsive coating exhibits dynamically enhancing interfacial interactions. On this adaptive surface, cells trigger the activation of α5β1 integrin and Rac signaling pathways during early adhesion. As the MC converts to SP, the interaction between the ligand and integrin is reinforced, leading to the further activation of αvβ3 integrin and RhoA/ROCK signaling pathways in the subsequent phase (Figure 5C and D). By studying how cells respond to dynamic biophysical cues and promoting their mechanical sensitivity, this sequential process can enhance cellular mechano-transduction and promote the osteogenic differentiation of mesenchymal stem cells. These findings offer an innovative and suitable strategy for bone regenerative therapies.

Bone volume and quality are influenced by physiological potential, with bone’s piezoelectric properties playing a key role by generating signals that drive remodeling and repair in response to mechanical stress [190, 191]. There is close interdependence and interaction of the various electroactive and electrosensitive components of bone tissue, including cell membrane potential, voltage-gated ion channels, intracellular signaling pathways and cell surface receptors, together with various matrix components such as collagen, HA, proteoglycans and glycosaminoglycans. Exploiting these properties, piezoelectric materials are used in bone regeneration for their energy conversion and catalytic abilities [192]. Piezoelectric materials have dielectric crystals and asymmetric centers that could directly convert various primary forms of energy in the environment into secondary energy, such as convert mechanical energy into electricity energy [192]. They have the ability to deform with physiological movements and consequently deliver electrical stimulation to cells or damaged tissue without the need of an external power source [190].

**Figure 6.**
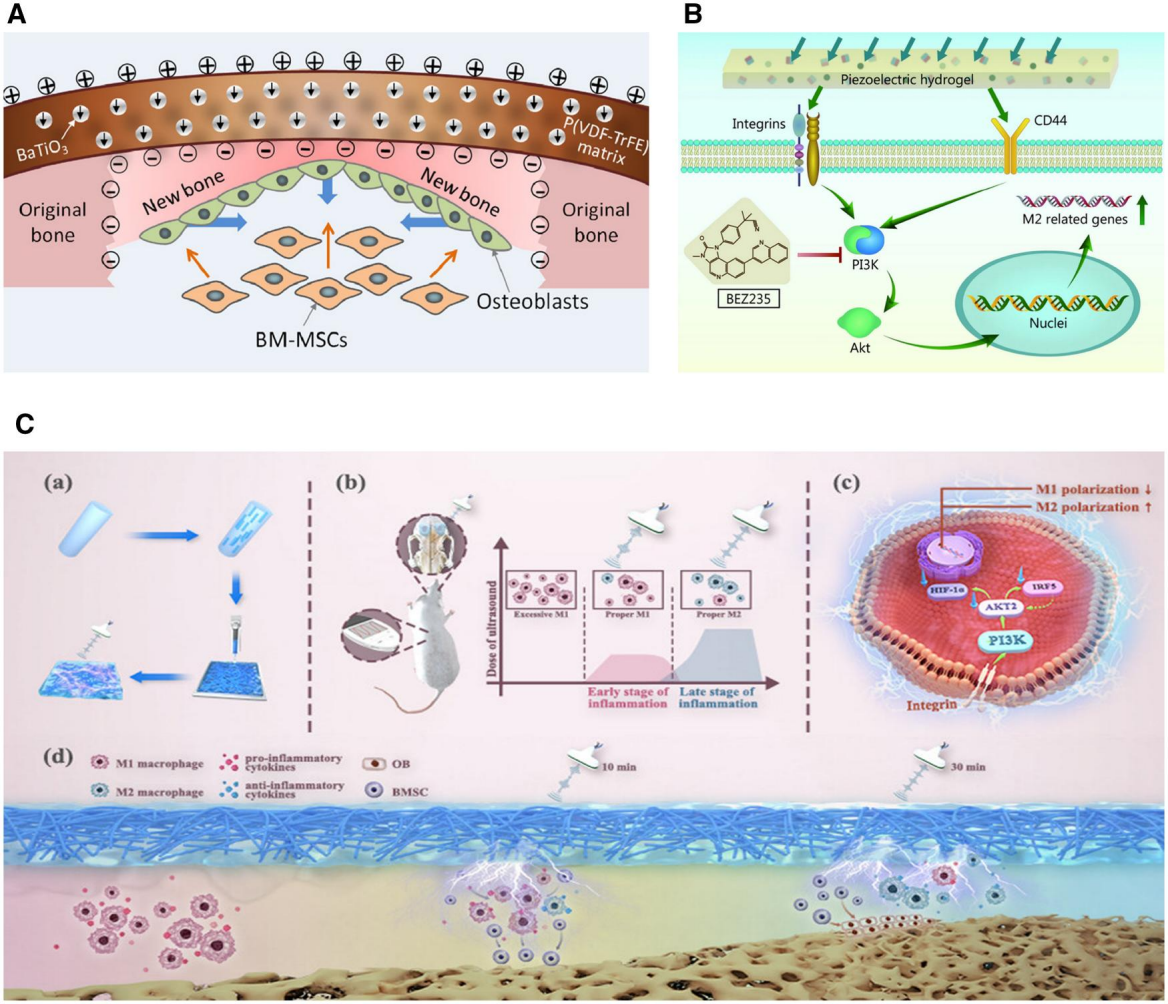
Piezoelectric properties and its applications. (**A**) Biomimetic electric microenvironment created by BTO NP/P(VDF-TrFE) composite membranes encouraging bone defect repair. Reproduced with permission from Ref. [195], Copyright 2016, American Chemical Society, Open Access. (**B**) The possible molecular mechanism by which piezoelectric hydrogel promotes bone repair by regulating macrophage M2 polarization and activating the PI3K/Akt axis. Reproduced with permission from Ref. [196], Copyright 2023, Military Medical Research, Open Access. (**C**) Temporal immunomodulation via wireless programmed electric cues achieves optimized diabetic bone regeneration. Reproduced with permission from Ref. [197], Copyright 2023, American Chemical Society.

Hence, biomimetic piezoelectric nanocomposite membranes have the potential to enhance the efficacy of GBR in dental clinics [193]. The utilization of a flexible piezoelectric membrane called P(VDF-TrFE) has been found to enhance osteogenic properties by modulating surface potentials. Specifically, when bone marrow mesenchymal stem cells are cultured on these membranes with a surface potential of −53 mV, they exhibit improved osteogenic properties, resulting in accelerated bone regeneration and the formation of mature bone structures [194]. The biomimetic electric microenvironment formed by BTO NP/P(VDF-TrFE) composite membranes has been proved to promote the repair of bone defects. Following corona poling treatment, the electrical dipoles of BTO NPs are realigned in the direction of the poling electric field, resulting in the generation of induced charges on the outer surface of the membrane. This phenomenon facilitates the recruitment of bone marrow-derived mesenchymal stem cells (BM-MSCs) through galvanotaxis and induces their differentiation into osteoblasts [195]. Deng *et al.* [195] developed a flexible nanocomposite membrane that mimics the endogenous electric potential (Figure 6A). By optimizing the composition ratio and applying corona poling treatment, the membrane can match the level of endogenous biopotential, thereby improving bone defect repair efficiency. Furthermore, the polarized nanocomposite membranes can modulate macrophage polarization through electrical signaling cues [198]. A piezoelectric hydrogel bone scaffold was made with a chitosan/gelatin matrix and polydopamine-modified ceramic HA and barium titanate nanoparticles (Figure 6B) [196]. It broadly induced macrophage polarization, facilitated HUVEC migration, tube formation and angiogenic differentiation, and facilitated osteo-differentiation and ECM mineralization. Furthermore, Sun’s [197] work combined piezoelectric effects with external field response (Figure 6C). Electric signals that can be controlled and programmed wirelessly, and are transmitted through ultrasound from a piezoelectric membrane, are able to regulate the timing of macrophage polarization. This process aligns with the pattern of immunoregulation observed during the natural healing process after implantation and effectively enhances the repair of diabetic bone. It is worth to note that piezoelectric materials have the capability to emit electrons/holes and catalyze redox reactions of substrates, thereby influencing biological processes. This phenomenon is referred to as piezo catalysis [199]. Currently, piezo catalytic materials are extensively utilized in medical applications such as tumor treatment, antisepsis, organic degradation, tissue repair and regeneration.

### Modulation of oral microbial homeostasis

The oral-maxillofacial bone defects are unique due to the presence of a microbial environment. The oral cavity hosts a complex, dynamic microflora where a balance between beneficial and harmful bacteria sustains periodontal health [200]. Dysbiosis, characterized by the rise of inflammation-associated bacteria and a more virulent microbiota, can disrupt this balance, leading to periodontitis and the deterioration of tooth-supporting structures [201]. An example of such an imbalance is the increased presence of *Actinobacillus actinomycetemcomitans*, which heightens the risk of periodontal damage if the regulatory systems cannot preserve the microbial equilibrium [202].

Thus, managing oral cavity infections demands a strategy that extends beyond mere bacterial eradication. It necessitates adjusting the host–microbial interaction and maintaining the oral microbiome balance. The aim is to remove pathogenic species and preserve or encourage commensal or beneficial ones [203]. Regrettably, the predominant approach for addressing oral microbial diseases at present is empirical antibacterial therapy. This method involves the widespread elimination of both harmful pathogens and beneficial microorganisms. Existing bone repair materials primarily emphasize anti-inflammatory or broad-spectrum antibiotic properties without considering the significance of preserving a balanced microbiota.

Various targeted strategies can be employed to eradicate particular pathogens and restore balance to the oral microbiome. One such approach is Probiotic Therapy, which involves the use of probiotics to rebalance the oral microbiome. Probiotics, when incorporated into mouthwash, lozenges or gum, can effectively combat harmful bacteria, influence metabolism, and modulate immune responses [204]. For example, lactobacilli have been found to disrupt *A.actinomycetemcomitans* colonization of the oral cavity by reducing its ability to interact with gingival epithelial cells and modulating cellular response [205]. Additionally, certain probiotic strains, such as BLIS K12 and BLIS M18, possess the natural ability to colonize the oral cavity and produce bacteriocins and bacteriocin-like inhibitory substances that suppress harmful pathogens [206]. Furthermore, *Akkermansia muciniphila*, a critical probiotic isolated from the human intestine, has been shown to specifically inhibit the effects of *Fusobacterium nucleatum* and alleviate periodontitis [207]. Emerging techniques, such as targeted antimicrobial peptides, aim to selectively neutralize harmful bacteria without disrupting the balance of the oral microbiome [208, 209]. Targeted interventions that focus on eradicating *Porphyromonas gingivalis* and collapsing its inflammation-associated flora offer novel strategies for periodontitis therapy [210]. It is also possible to prevent periodontitis by targeting specific pathogens in advance. For instance, treatment with inactivated *P.gingivalis* prior to periodontal infection induced specific antibodies against *P.gingivalis* and protected mice from periodontitis-induced dysmetabolism [211]. Moreover, a metagenomic approach has been utilized to analyze antibiotic-resistant genes and metal-resistant genes in dental plaque, providing valuable insights into the distribution of these genes in the oral microbiota of periodontitis patients and potentially improving the efficacy of treatment regimens [212].

### Interplay of osteo-immune responses in oral-maxillofacial bone regeneration

The oral microenvironment is susceptible to bacterial infections, which invoke both innate and adaptive immune responses, including actions by phagocytes, beta-defensin and the complement system [213, 214]. Additionally, dental materials may trigger immune responses, causing inflammation or rejection, possibly leading to fibrotic tissue proliferation and alveolar bone resorption [215]. In order to enhance integration and long-term efficacy, the capacity of biomaterial to elicit a favorable immune response is of importance.

**Figure 7.**
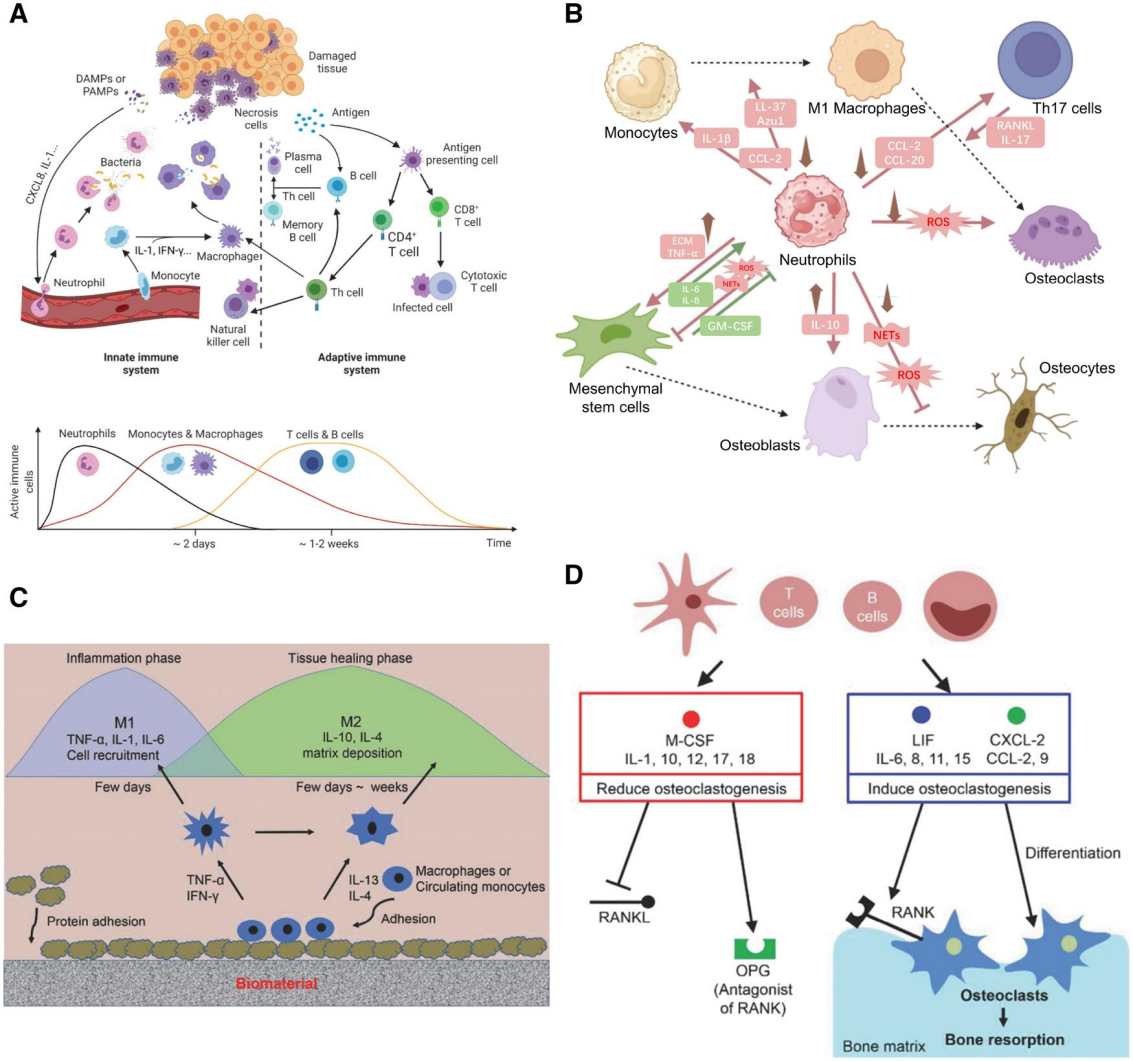
Immune cells and bone regeneration. (**A**) Overview and kinetics of innate and adaptive immune system participate in immune response. Reproduced with permission from Ref. [87], Copyright 2021, Wiley. (**B**) Neutrophils influence the osseointegration process by releasing factors that affect the activation of macrophages and T cells, as well as interacting with osteoblast cell lines [216] (the arrows show how neutrophils release inflammatory factors to improve osseointegration). (**C**) General schematic of the biomaterial modulation of macrophage response in tissue healing. (**D**) Cross-talk among T cells and B cells, osteoblasts and osteoclasts during bone healing processes. Reproduced with permission from Ref. [217]. Copyright 2018, Wiley.

Immune cells could release regulatory molecules that lead to an imbalance between osteoclasts and osteoblasts (Figure 7), resulting in osteolysis, osteoporosis, etc. [85]. For example, mature neutrophils, FcgR1hi and differentiated immunomodulatory macrophages indicate favorable osseointegration while the increased immature neutrophils, Ly6C^+^CCR2^hi^ monocytes and S100a8hi macrophages induce the formation of a fibrous capsule around the stainless steel implant [218].

Different immunomodulatory methods could target different immune cells like Neutrophils, Monocytes, Macrophages and T lymphocytes (Figure 7A) [219]. During period of acute inflammation, junctional epithelial cells express chemotactic factors that recruits influx of neutrophils [213]. Subsequently, the role of neutrophils in bone regeneration may be varied (Figure 7B). Neutrophils could lead to the production of ROS [219], or be N2-polarized, which then secrete stromal cell-derived factor-1α (SDF-1α) for bone mesenchymal stem cell (BMSC) [220]. Some believe that materials should reduce neutrophil recruitment at the surgery site to provide anti-inflammatory properties [221]. Some are working to manipulate inflammation by regulating its recruitment and then apoptosis. A pH-responsive hydrogel enables burst release of formyl-met-leu-phe (fMLP) to rapidly recruit neutrophils for heightened inflammation initiation and then expose SiO_2_-FasL to activate neutrophils apoptosis via FasL-Fas signaling, triggering timely inflammation resolution [222]. Some also promote neutrophils to exert the anti-inflammatory function. A nano-layer composed of PEO-FeZn suppresses the expression of genes associated with immune evasion and facilitate the generation of neutrophil extracellular traps by inducing the extracellular release of DNA fibers and granule proteins, and thereby suppressing bacterial invasion and promoting osseointegration *in vivo* [223].

The role of macrophage polarization in the pathogenesis and treatment of alveolar bone defect is pivotal [224, 225]. Macrophages are the main source of inflammatory factors involved in periodontal tissue destruction [224]. It is commonly agreed that a timely switch from M1 macrophage phenotype of early stage to M2 macrophage phenotype results in an osteogenic cytokine release, while a prolonged M1 polarization phase leads to an increase in fibrosis-enhancing cytokine release (Figure 7C) [85]. Numerous researchers are working to suppress M1 macrophage polarization and activate M2 macrophages to promote osteogenesis [226–229]. There is more to the classification of macrophages than M1 and M2. Study suggests that CD301b^+^ macrophages aid in periodontal bone repair and an osteogenic inducible nano-capsule (OINC) first absorbs pro-inflammatory cytokines and then releases IL-4, which promotes CD301b^+^ macrophage enrichment, thus enhances periodontal bone regeneration [139].

Specialized subsets of T lymphocytes such as CD4^+^ T cells play roles in immune response and autoimmune diseases [230]. The balance between regulatory T-cell (Treg) and T helper cell 17 determines the direction of the inflammatory response [231]. Immunomodulators can regulate regulatory T cells (Tregs), responsible for maintaining immune tolerance and preventing autoimmunity. By augmenting Treg populations, immunomodulators instill restraint within the immune response, preventing hyperactivity and inflammation [219]. Limited research has focused on Treg cells to promote periodontal bone repair. However, Liu *et al.* [232] have identified a novel approach involving the localized release of IL-2/TGF-β and miR-10a to recruit T cells and induce their transformation into Tregs, and demonstrated promising outcomes in Treg-mediated immunotherapy for the prevention of bone loss.

In addition to immune cells, the immunomodulatory effects of DSCs have been mentioned above [143]. A PCL/LAP nanofibrous membrane boosted the growth and bone-forming transformation of PDLCs, leading to the promotion of anti-inflammatory N2 neutrophil production and M2 macrophage polarization, ultimately expediting periodontal regeneration in living organisms [233].

It is worth mentioning that uncontrolled anti-inflammatory measures may not promote bone regeneration, for excessive dampening of the inflammatory/immune response can delay fracture healing or lead to non-union [234]. Furthermore, inhibiting inflammatory pathways can result in the systemic transmission of oral bacteria in rats [235]. In this regard, an improved understanding of molecular interactions and mechanisms associated with innate and adaptive immune responses will aid the development of new periodontal therapeutics. Materials could be capable of precisely releasing cytokines and inflammatory factors in line with the natural fluctuation of these biomolecules within the bone defect [236]. Thus, the development of intelligent controlled-release materials is highly practical.

### Smart stimuli-responsive biomaterials

As early as 1998, *Nature* highlighted the advantages and significance of local sustained release in drug administration [237]. In comparison to traditional methods such as oral administration and injection, *in situ* local administration offers prolonged efficacy, targeted tissue response and optimal bioavailability [238] Nowadays, the development of smart stimuli-responsive biomaterials is flourishing to achieve better sequential and spatiotemporal release of loaded cargo. The specific mechanisms, clinical applications and limitations of smart stimuli-responsive biomaterials for bone therapeutics and regeneration have been comprehensively summarized by researchers [236]. Typical bio-responsive actions of the stimuli-triggered responses are detailed and summarized in another review [239]. Hydrogels are excellent carriers for various cargoes, including living cells and GFs, due to their high permeability, water retention and strong loading capacity [240], thus have emerged as a major material for intelligent controlled release. Amirthalingam and his coworkers [241] have extensively studied stimuli-responsive hydrogel systems, including their recent developments, fabrication strategies, internal principles and applications.

**Figure 8.**
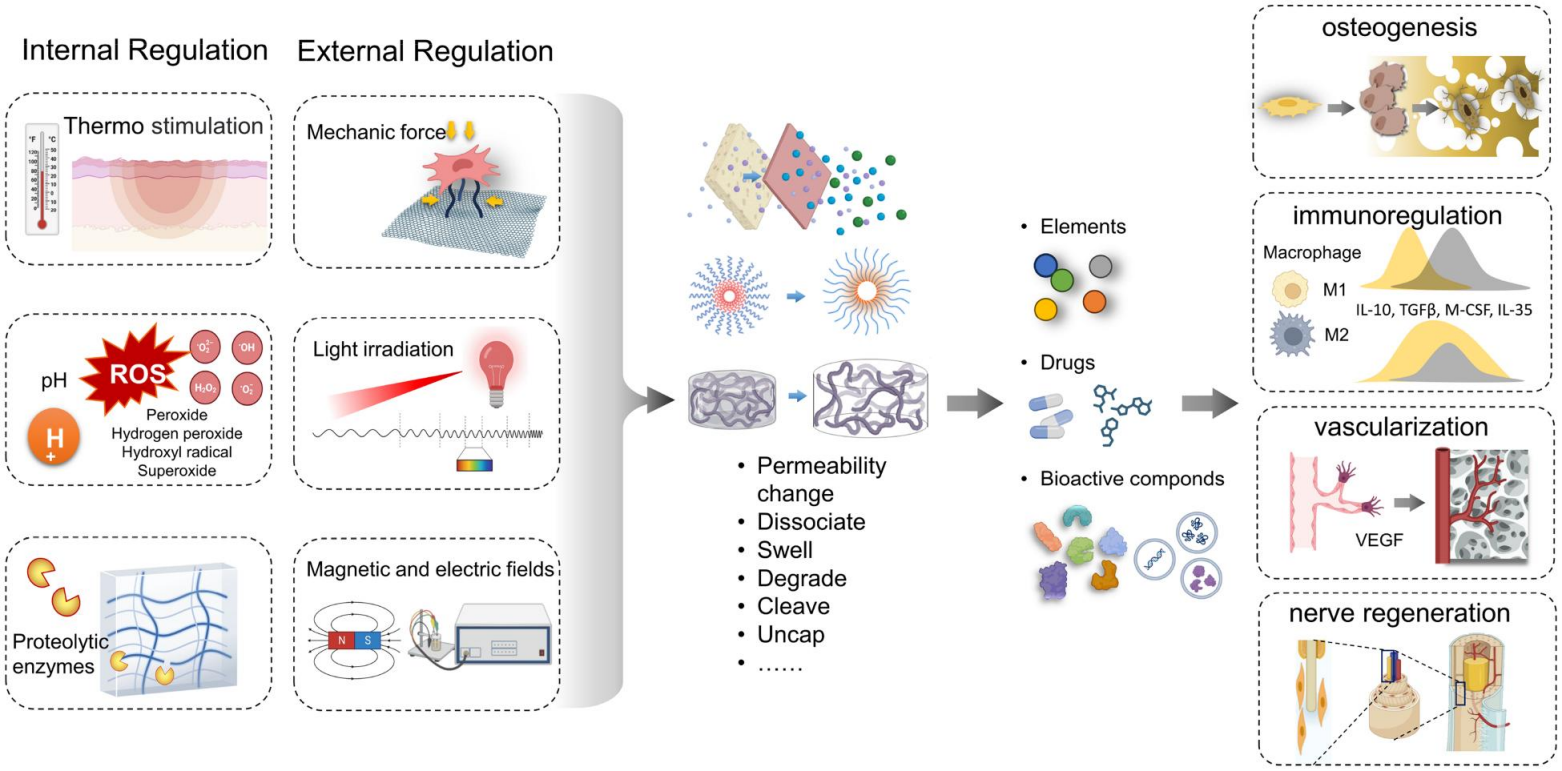
Smart stimuli-responsive biomaterials can be triggered by internal and external stimulation to release their loaded elements, drugs, molecules or other bioactive components, thereby facilitating bone formation, immunomodulation, angiogenesis and nerve regeneration. Created with BioRender.com.

Present drug delivery approaches emphasize controlled, responsive release, activated by external or internal stimuli (Figure 8) [50, 236]. Internal regulation refers to the response of materials to stimulation that occurs within the body. This stimulation can be physical, chemical and biological. Physical stimulation could be thermal energy, changes in skin impedance, etc. [242–244]. Thermo-sensitive properties have a wide range of applications, including thermotherapy for cancer treatment and palliation of painful bone metastases [245–248]. In the context of bone healing, thermos-sensitive materials have also been developed [249]. This material utilizes host–guest interactions between CP5 rings and A stalks on Zr-MOFs, which are weakened as the temperature increases. This leads to the disassociation of the rings from the MOF surfaces, unblocking the pores and gradually releasing the stored cargo molecules. Thermal-dependent deformation is another application scenario. A smart thermo-sensitive hydrogel was developed [243], the hydrogel is capable of producing thermo-related deformation, thus better fit into bone defect wounds, and then allowing for a burst release of PDGF-BB and a sustained release of BMP-2.

Chemical stimulation, such as changes in pH or the redox environment, can trigger internal regulation. Tissue injury can lead to the production of reactive oxygen species (ROS), causing oxidative stress, inflammation and cellular damage [250]. In the context of bone healing, a few studies have explored the use of redox-sensitive drug delivery methods. For instance, a layer-by-layer assembly-compatible polycation that degrades in response to ROS generated by cells can enable targeted drug release. Thioketal-based polymers, which can be selectively cleaved by physiological levels of ROS, have been used to achieve tunable release of therapeutic bone morphogenetic protein-2 (BMP-2) [251]. Another approach involves incorporating disulfide-containing PEG-based scaffolds with rhBMP-2, where oxidative stress-induced glutathione triggers scaffold degradation and promotes bone healing [50]. Additionally, changes in pH or other microenvironmental indicators can also trigger drug release. For example, a scaffold designed to repair alveolar defects can respond to acidic pH conditions, leading to rapid hydrolysis and the release of TNFα antibodies to regulate inflammation [252].

Biological stimulation, such as changes in blood glucose levels or the presence of proteolytic enzymes like MMP and thrombin [253, 254], can also drive internal regulation. Controlled release materials that respond to individual-specific molecules as triggers are particularly sensitive. For instance, materials that respond to changes in blood glucose levels can be used to repair periodontitis bone defects in diabetic patients, the examples will be demonstrated below. Similarly, the changes of MMP are also remarkable in the periodontal immune microenvironment, so it has certain research and application value. MMP-8 is one of the most important biomarkers for periodontitis, thus the MMP-8-responsive strategy is fit for treating chronic periodontitis and peri-implantitis. A hydrogel was synthesized from diacrylate-containing polyethylene glycol-based scaffolds and a cysteine-terminated peptide crosslinker, which can be cleaved by MMP-8, and the MMP-8-responsive release behavior of the on-demand drug delivery system was evaluated *in vitro* [254]. In addition, since thrombin-responsive systems possess high sensitivity and fast response rates, a thrombin-cleavable peptide is introduced as a linker during the conjugation of the drug to the main chain of hyaluronic acid (HA) [253]. The peptide can be cleaved when thrombin is activated, triggering the release of the drug from the HA scaffold.

External stimuli primarily consist of physical factors such as mechanics, light, electric/magnetic/electromagnetic fields and ultrasonics [241]. It has been observed that physical stimulation itself can enhance osteogenesis and reduce inflammation during bone regeneration [255]. Near-infrared (NIR) light irradiation, for example, has the advantage of deeper tissue penetration compared to UV and visible light [241]. NIR stimulation itself has been found to promote osteoblast differentiation and accelerate bone regeneration [256]. Triggering drug administration on this basis can double effectiveness. A NIR-activable scaffold (CBP/MBGS/PTHrP-2) with photothermal agent-doped is developed to promote osteogenesis and angiogenesis for high-efficacy bone regeneration [257]. Upon on/off NIR irradiation, the thermoresponsive hydrogel gating undergoes a reversible phase transition to allow precise control of dual-mode PTHrP-2 (a PTH derivative) release capability. Rifaie-Graham and his research team have proposed a novel nanosystem that is responsive to light [258]. This nanosystem consists of a combination of enzyme-containing polymersomes, which are capable of light-controlled chemical communication, adjustable feedback and connection to macroscopic oscillations. The polymersome membrane, containing donor-acceptor Stenhouse adducts, becomes permeable when exposed to green light. This permeability activates esterase activity and reduces the pH level, thereby initiating non-equilibrium communication within the nanosystem.

Magnetic and electric fields are currently being explored for controlled drug delivery strategies. As for magnetic field, it is reported that an external static magnetic field with magnetic responsive scaffolds incorporated with magnetic nanoparticles effectively promotes osteogenic differentiation and neogenesis of endotheliocytes *in vitro* and *in vivo* [259]. As for electromagnetic field, it is reported that an external magnetic field force conduct on the core of material increase charge density on the shell and finally increase the membrane surface potential, hence activates osteogenesis and bone regeneration [260]. There are also liposome-rhBMP-2 nanocomplexes that release rhBMP-2 in response to non-thermogenic clinical diagnostic ultrasound exposure [261].

To conclude, existing response strategies include the following approaches: (i) dynamically adjusting the drug release based on variations in the microenvironment, with a greater emphasis on drug release when environmental indicators exhibit more pronounced changes; (ii) implementing a dual-response hierarchical regulation system that effectively addresses multiple environmental alterations simultaneously [262]; (iii) employing sequential delivery techniques, such as promoting inflammation during the early stages and subsequently inhibiting it, to better align with the microenvironmental requirements for bone regeneration; (iv) adopting spatially controlled delivery, which enables physiologically relevant release profiles and spatial gradients that closely mimic natural healing responses; and finally, (v) utilizing a logic-based diagnostic and therapeutic hydrogel with multi-stimuli responsiveness to orchestrate bone regeneration [263].

Traditional drug-release materials lack a strategic approach as they degrade, resulting in slow drug release and passive drug release curves. The optimization in recent years mainly lies in (i) the development of new response mechanisms to achieve high selectivity and sensitivity; (ii) achieving improved spatial and temporal control over dosage and delivery of bioactive molecules within living organisms, thereby facilitating precise modulation of their biological functions; (iii) the last but most important one, verification of long-term stability and responsiveness with satisfactory safety profiles, especially in the highly complex *in vivo* milieu [239, 241, 264].

### Multifaceted approach for comorbid condition management

Numerous oral diseases are intricately linked to and may even exacerbate systemic ailments, including but not limited to diabetes, metabolic syndrome, obesity, eating disorders, liver disease, cardiovascular disease (CVD), Alzheimer’s disease, rheumatoid arthritis, adverse pregnancy outcomes and cancer [265–271]. Research has focused on investigating the interaction between oral diseases and various systemic diseases, and creating a range of treatment methods. Given that the oral-maxillofacial restoration of comorbid patients is more challenging than that of healthy individuals, devising a multifaceted repair strategy for the former can also offer valuable insights into treating the latter group.

One such approach that has been widely utilized is the bone repair strategy for diabetes. Diabetes and periodontitis are complex chronic diseases with a bidirectional link. Patients with diabetics are more prone to periodontitis, due to high glucose levels worsening periodontal tissue integrity. The environment marked by elevated glucose levels, proteinase activity, inflammatory state, low pH, high concentration of ROS and advanced glycation end products (AGE) mutually aggravates the conditions, impairing bone regeneration. Concurrently, systemic inflammation (as indicated by acute-phase and oxidative stress biomarkers) is heightened due to the entry of periodontal organisms and their virulence factors into the bloodstream [272]. Consequently, severe periodontitis can detrimentally impact glycemic control in both diabetic and non-diabetic patients. A current emerging trend focuses on combining locally diabetic-responsive drugs with bone repair materials to target the local microenvironment for anti-inflammatory purposes, promoting bone growth and vascular regeneration.

**Figure 9.**
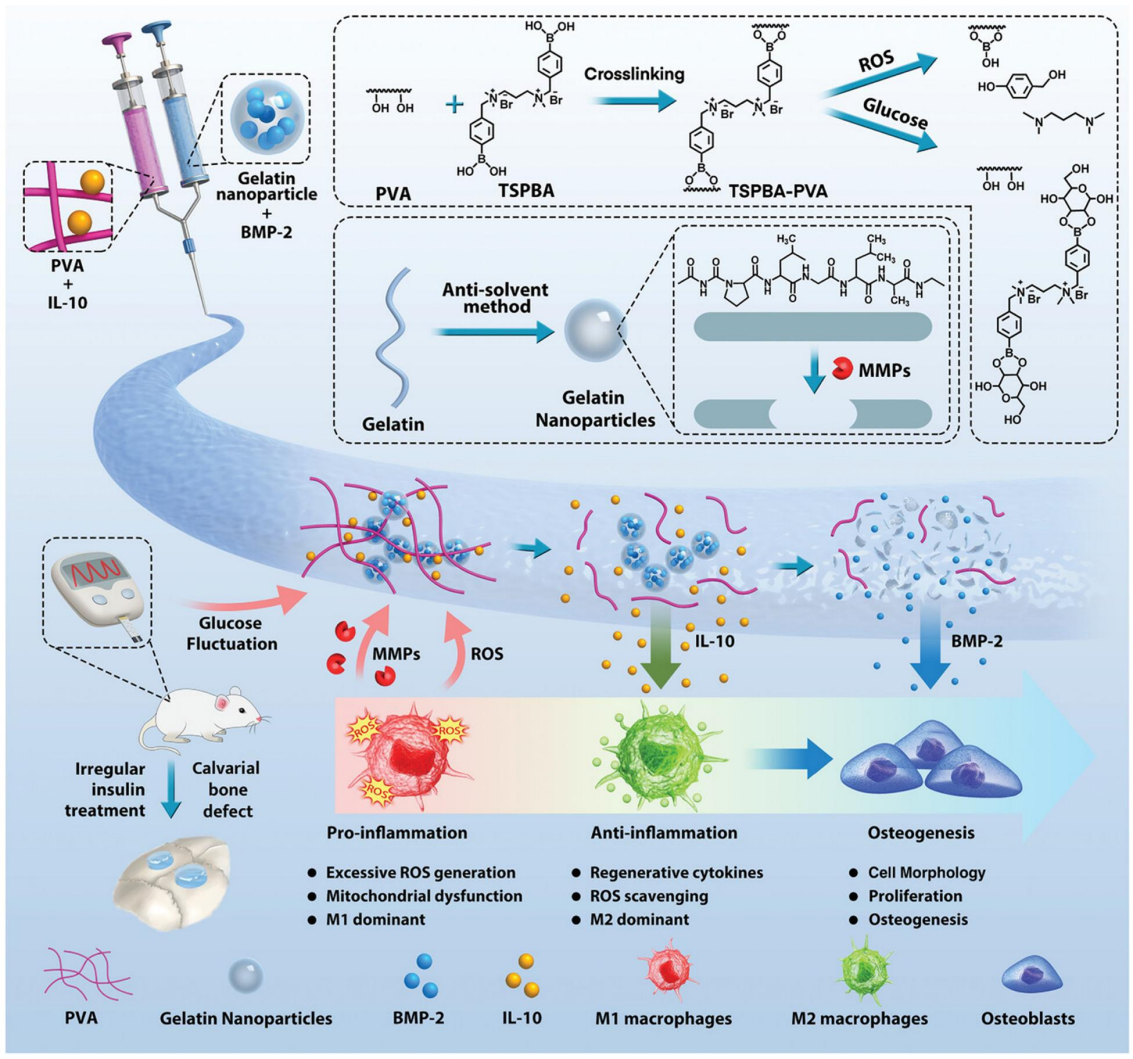
A logic-based hydrogel with multi-stimuli responsiveness orchestrates diabetic bone regeneration through diagnostic and therapeutic functions. The double-network hydrogel is composed of a primary network of PVA crosslinked with PBA, and a secondary network of gelatin colloids. Exposing to glucose, ROS and MMPs would initiate the cleavage of phenylboronic ester bonds, leading to the degradation of the PVA network and the initial release of IL-10, thus to regulate immune response in the first place. Later, BMP-2 housed within the gelatin network, which are encased by the PVA network, can be controlled release at a subsequent stage, thus to achieve osteogenesis. Reproduced with permission from Ref. [263], Copyright 2023, Wiley.

The existing responsive mechanisms suitable for bone defect repair in the context of diabetes include (i) glucose-binding molecule (particle)-based systems, (2) GOx-based systems responsive to pH, hypoxia, and H_2_O_2_, as well as (3) phenylboronic acid (PBA)-based systems [126, 262, 273, 274]. These advancements hold great potential in addressing the challenges associated with diabetic bone repair. The current approaches for repairing bone defects in diabetic patients are limited in terms of specificity and strategy. Li [263] put forward a ‘diagnostic’ and therapeutic dual-logic-based hydrogel (Figure 9). The ‘diagnostic’ function could determine when to release what kind of drugs in a diabetic microenvironment by responding to specific pathological cues (glucose fluctuation, ROS, MMPs). The therapeutic logic could program the sequential release of different drugs to achieve immune regulation in the first stage and osteogenesis in the last stage for better tissue regeneration.

Beyond alveolar bone repair in diabetic patients, the treatment of other comorbid conditions awaits further investigation. Dysbiosis of the oral microbiome and intestinal diseases may be interrelated and mutually influential since oral and gut microbiomes could regulate physiological functions and pathological processes interdependently [275]. For instance, *P.gingivalis*, which disrupts the subgingival microbiome and immune defense, can also lead to dysregulation in the gut [276, 277]*.* Meanwhile, periodontal therapy such as usage of prebiotics and probiotics may alleviate gastrointestinal disorders induced by oral bacteria [278]. Hence, when addressing alveolar bone defects caused by periodontitis, an integrated treatment for both periodontitis and gastrointestinal diseases could be considered simultaneously [278]. Apart from that, owing to interactions between the oral microbiome and cardiovascular inflammation, there is robust epidemiologic evidence linking periodontitis with heightened cardiovascular disease (CVD) risk [279, 280]. Oral dysbiosis could lead to CVD through various mechanisms, such as immunomodulation, endothelial dysfunction, molecular mimicry, antibody cross-reactivity, protein citrullination, etc. Thus, addressing oral dysbiosis during periodontitis bone repair may be significant in CVD prevention and management [279]. However, research on related integrated therapies is limited and has not received equal attention as a diabetic condition.

## Conclusion and perspectives

The demand for oral-maxillofacial bone regeneration is growing progressively year after year, especially for periodontal and alveolar bone. Periodontal damage leads to multi-walled bone defects, making alveolar bone regeneration more challenging, thus require bone grafts with high osteogenic potential. The defects here are usually highly irregular, so 4D printing should be utilized to fabricate a shape-memory scaffold to close-fit these irregular defects. Meanwhile, the soft tissue remodeling here is rather rapid and dynamic, so it’s pivotal to use the membrane to prevent epithelia attachments and soft tissue ingrowth during alveolar bone regeneration. Throughout this entire process, the oral environment is highly exposed to bacteria and infection, thus regulation around microbial homeostasis and osteo-immune is important. We present an overview of current bone repair materials and found a lot of potential for enhancement in addressing the specific requirements of oral-maxillofacial bone repair.

Subsequently, we conclude the current modifications of oral-maxillofacial bone regeneration materials, such as mechanical properties, surface topography, pore design and integration of bioactive components, and put forward limitations of these modifications. It is necessary to match the mechanical requirements under different restoration scenarios and adapt to the dynamic mechanical characteristics during bone remodeling. Modifications around the surface and pore structure should consider the antibacterial and immunomodulatory needs of the oral-maxillofacial environment. In the future, we should explore and fully understand the exact mechanisms of bioactive components to highly effectively design and develop the next generation of oral-maxillofacial bone regeneration materials.

Importantly, we highlight strategies including the manipulation of the physical microenvironment, modulation of oral microbial homeostasis, regulation of osteo-immune responses, application of smart stimuli-responsive biomaterials and management of comorbid conditions. Future research should integrate innovative ideas from bone repair while focusing on the unique attributes of oral-maxillofacial bone and periodontal tissue to explore a multitude of possibilities. For instance, managing a balance of microbial homeostasis and osteo-immune responses. Or else, conducting the multifaceted repair strategy of comorbid conditions.

In summary, we outline the existing classifications, modifications, the future developments of oral-maxillofacial bone regeneration materials, especially for periodontal and alveolar bone, from the perspective of anatomical structure, environmental characteristic and regeneration requirements. This review may provide practical insights to researchers in the field of both oral medicine and biomaterial science.
